# Telomere Length Trajectory and Its Determinants in Persons with Coronary Artery Disease: Longitudinal Findings from the Heart and Soul Study

**DOI:** 10.1371/journal.pone.0008612

**Published:** 2010-01-08

**Authors:** Ramin Farzaneh-Far, Jue Lin, Elissa Epel, Kyle Lapham, Elizabeth Blackburn, Mary A. Whooley

**Affiliations:** 1 Division of Cardiology, San Francisco General Hospital, San Francisco, California, United States of America; 2 Department of Medicine, University of California San Francisco, San Francisco, California, United States of America; 3 Department of Biochemistry and Biophysics, University of California San Francisco, San Francisco, California, United States of America; 4 Department of Psychiatry, University of California San Francisco, San Francisco, California, United States of America; 5 Veterans Affairs Medical Center, San Francisco, California, United States of America; 6 Department of Epidemiology and Biostatistics, University of California San Francisco, San Francisco, California, United States of America; University of Valencia, Spain

## Abstract

**Background:**

Leukocyte telomere length, an emerging marker of biological age, has been shown to predict cardiovascular morbidity and mortality. However, the natural history of telomere length in patients with coronary artery disease has not been studied. We sought to investigate the longitudinal trajectory of telomere length, and to identify the independent predictors of telomere shortening, in persons with coronary artery disease.

**Methodology/Principal Findings:**

In a prospective cohort study of 608 individuals with stable coronary artery disease, we measured leukocyte telomere length at baseline, and again after five years of follow-up. We used multivariable linear and logistic regression models to identify the independent predictors of leukocyte telomere trajectory. Baseline and follow-up telomere lengths were normally distributed. Mean telomere length decreased by 42 base pairs per year (p<0.001). Three distinct telomere trajectories were observed: shortening in 45%, maintenance in 32%, and lengthening in 23% of participants. The most powerful predictor of telomere shortening was baseline telomere length (OR per SD increase = 7.6; 95% CI 5.5, 10.6). Other independent predictors of telomere shortening were age (OR per 10 years = 1.6; 95% CI 1.3, 2.1), male sex (OR = 2.4; 95% CI 1.3, 4.7), and waist-to-hip ratio (OR per 0.1 increase = 1.4; 95% CI 1.0, 2.0).

**Conclusions/Significance:**

Leukocyte telomere length may increase as well as decrease in persons with coronary artery disease. Telomere length trajectory is powerfully influenced by baseline telomere length, possibly suggesting negative feedback regulation. Age, male sex, and abdominal obesity independently predict telomere shortening. The mechanisms and reversibility of telomeric aging in cardiovascular disease deserve further study.

## Introduction

Telomeres are tandem repeat DNA sequences (TTAGGG)_n_ which form a protective cap at the ends of eukaryotic chromosomes[1]. During somatic cell division, DNA polymerase cannot fully replicate the 3′ end of linear DNA, thus resulting in an obligate and progressive loss of telomeric repeats[2]. After a critical degree of telomere shortening, cells lose the ability to replicate and may cease dividing (senescence) or undergo programmed cell death[3]. Human telomere length is influenced by both genetic and environmental factors[4]. These fundamental observations have led to increasing interest in telomere length as the possible basis for a “biological clock” which integrates the cumulative lifetime burden of genetic factors and environmental stressors independently of chronological age[5].

A growing body of evidence has demonstrated an independent association between short telomere length and cardiovascular events, including myocardial infarction[6], [7], congestive heart failure[8], and death[9], [10]. In addition, several large epidemiologic studies have demonstrated cross-sectional associations of short telomeres with risk factors for coronary artery disease[11], [12], [13]. However, the natural history of telomere length, and the determinants of temporal change in telomere length in persons with established cardiovascular disease, have not previously been investigated.

We sought to evaluate the trajectory of change in leukocyte telomere length, and to identify the demographic, clinical and biochemical predictors of telomere trajectory, in a prospective cohort study of patients with stable coronary artery disease. In particular, we aimed to detect modifiable risk factors, which influence telomere length over time, and which may lead to novel insights into the mechanisms of biological aging.

## Methods

### Participants

The Heart and Soul Study is a prospective cohort study investigating the influence of psychosocial factors on cardiovascular events in stable coronary artery disease. The enrollment process has been previously described[14]. Eligible participants were recruited from outpatient clinics in the San Francisco Bay Area if they met at least one of the following inclusion criteria: 1) history of myocardial infarction, 2) angiographic evidence of at least 50% stenosis by area in at least one coronary artery, 3) evidence of exercise-induced ischemia by treadmill electrocardiogram or stress nuclear perfusion imaging, or 4) history of coronary revascularization. Individuals were excluded if they had a history of myocardial infarction in the past 6 months, deemed themselves unable to walk 1 block, or if they were planning to move out of the local area within 3 years.

The study protocol was approved by: the University of California San Francisco Committee on Human Research, the Research and Development Committee at the San Francisco VA Medical Center, the Medical Human Subjects Committee at Stanford University, the Human Subjects Committee at the VA Palo Alto Health Care System, and the Data governance Board of the Community Health Network of San Francisco. All participants provided written informed consent. Between September 2000 and December 2002, a total of 1024 participants enrolled in the study. Of these, 954 provided DNA samples for analysis at the baseline visit, and 608 also provided DNA samples after 5 years of follow-up.

### Telomere Length Assay

Blood samples collected from participants at baseline and follow-up visits were subjected to density gradient centrifugation to yield a buffy coat preparation containing peripheral blood leukocytes and stored at −70C. Genomic DNA was isolated according to standard procedures using the Wizard Genomic DNA Purification Kit (Promega Corp, Madison WI). Purified DNA samples were diluted in 96-well microtiter source plates to a fixed concentration of 3 ng/ul. Relative mean telomere length was measured from DNA by a quantitative polymerase chain reaction (qPCR) assay that compares mean telomere repeat sequence copy number (T) to a reference single copy gene copy number (S) in each sample as previously described and validated by comparison with Southern blot terminal restriction fragment (TRF) analysis[15]. Standard curves were derived from serially diluted reference DNA. The T/S ratio was determined from the average quantity of reference DNA found to match with each experimental sample for the copy number of the targeted template (the number of telomere repeats for T and the number of beta-globin gene copies for S).

The primers for the telomere qPCR were tel1b [5′-CGGTTT(GTTTGG)5GTT-3′] and tel2b [5′-GGCTTG(CCTTAC)5CCT-3′], each used at a final concentration of 900 nM. Human beta-globin qPCR primers were: hbg1 [5′-GCTTCTGACACAACTGTGTTCACTAGC-3′], used at a final concentration of 300 nM, and hbg2 [5′-CACCAACTTCATCCACGTTCACC-3′], used at a final concentration of 700 nM. The final reaction mix was: 20 mM Tris–HCl, pH 8.4; 50 mM KCl; 200 nM each dNTP; 1% DMSO; 0.4×Sybr Green I; 44 ng Escherichia coli DNA; 0.8 U Platinum Taq DNA polymerase (Invitrogen) per 11 µl reaction; 10 ng genomic DNA. All PCRs were carried out on a Roche Lightcycler 480 real-time PCR machine (Roche Applied Science, Indianopolis, IN).

To control for inter-assay variability, eight control DNA samples were included in each run. The T/S ratio of each control DNA was divided by the average T/S for the same DNA from each run to obtain a normalizing factor. The average normalizing factor across all eight samples was then used to adjust the participant DNA measurements to obtain the final T/S ratios in each batch. The coefficient of variability for the eight control samples across all batches was 6%. The T/S ratio at baseline and follow-up for each participant was measured in duplicate. When the duplicate T/S value and the initial value varied by more than 7%, the sample was run for a third time, and the two closest values were used. Approximately 15% of samples required assay in triplicate. Using this method, the inter-assay coefficient of variability for telomere length measurement was 3.7% (equivalent to 0.20 kilobases with respect to the baseline mean). The intra-assay coefficient of variability was 2.5% (equivalent to 0.13 kilobases with respect to the baseline mean).

To determine the conversion factor for the calculation of approximate base pair telomere length from T/S ratio, the above method was used to determine the T/S ratios, relative to the same reference DNA, for a set of genomic DNA samples from the human fibroblast primary cell line IMR90 at different population doublings, as well as with the telomerase protein subunit gene (hTERT) transfected into a lentiviral construct. The mean TRF length from these DNA samples was determined using Southern blot analysis, and the slope of the plot of mean TRF length versus T/S for these samples served as the conversion factor for calculation of telomere length in base pairs from the T/S ratio. The equation for conversion from T/S ratio to base pairs for this study was base pairs = 3274+2413*(T/S).

Measurement of leukocyte telomere length was performed in a blinded fashion without knowledge of the clinical data.

### Other Measurements

Baseline demographics, age, sex, and self-reported ethnicity, education, and income level were obtained by questionnaire. Cardiovascular co-morbidities and prior medical history were determined by self-report. Medication use was determined by having participants bring bottles to the study appointment during which study personnel recorded all medications. Participants were weighed and measured without shoes. Waist and hip circumferences were measured with a flexible plastic measure to the nearest 0.1-centimeter. Waist circumference was measured midway between the lower rib margin and iliac crest. Hip circumference was measured at the level of the greater trochanters. Waist-to-hip ratio was calculated as waist circumference divided by hip circumference. Body mass index was calculated as weight in kilograms divided by height squared in meters. Physical activity, defined as a dichotomous predictor (active versus inactive), was determined by questionnaire. Exercise capacity was measured at peak exertion during a symptom-limited exercise-treadmill stress test as previously described[16].

All patients underwent complete resting 2-dimensional echocardiography and Doppler examination using an Acuson Sequoia ultrasound system (Siemens Medical Solutions, Mountain View, CA) with a 3.5-MHz transducer. The left ventricular ejection fraction (LVEF) was calculated as (end diastolic volume – end systolic volume)/end diastolic volume.

Fasting venous blood samples were obtained at the baseline visit to measure serum biomarkers. Fasting glucose, HDL- and LDL-cholesterol levels, and C-reactive protein (CRP) were measured in a clinical laboratory setting. CRP was measured using the Roche Integra high-sensitivity assay (Roche, Indianapolis, Indiana) in 229 participants and (due to a change in the laboratory) the Beckman Extended Range high-sensitivity CRP assay (Beckman, Galway, Ireland) in the remaining participants. We used the “Human Serum Adipokine Panel A” immunoassay to measure adiponectin, and the “Human Serum Adipokine Panel B” to measure leptin, TNF alpha, and insulin (LINCOplex® system; Millipore, St Charles, MO). The R&D Systems (Minneapolis, MN) Quantikine HS IL-6 Immunoassay was used to determine the concentration of IL-6.

### Statistical Analyses

Baseline and follow-up telomere lengths were normally distributed. Continuous variables with a skewed distribution were natural logarithm transformed prior to further analysis. We categorized leukocyte telomere trajectory into three groups: shortened (defined as >10% decrease in telomere length), maintained (defined as ±10% change in telomere length), and lengthened (defined as >10% increase in telomere length). Differences in means and proportions of baseline characteristics by telomere trajectory were compared with the use of analysis of variance and the chi-squared test respectively. All p-values were two-tailed.

To identify the independent predictors of leukocyte telomere trajectory, we used multivariable linear regression with stepwise backward selection of candidate variables in Table 1. These predictors were chosen *a priori* based on reported cross-sectional associations with telomere length, biological plausibility, and established cardiovascular risk factors. Covariate selection was checked by visual inspection of directed acyclic graphs[17]. Variables were retained in the model at a significance level of p<0.1. Age, sex, ethnicity, and LVEF were retained in all models for face validity. For all linear regression models, the assumption of linearity was checked by visual inspection of component plus residual plots. The incremental contributions of polynomial terms in the predictors were evaluated by the likelihood ratio test. The normality assumption was checked by review of residual histograms and normal quantile-quantile plots. In order to identify mediating factors that may account for the observed associations of waist-to-hip ratio with telomere trajectory, we forced body mass index, adipokine levels and biomarkers of systemic inflammation back into the linear regression models.

**Table 1:** Baseline characteristics of study population categorized by trajectory of leukocyte telomere length over five years.

VARIABLE	Shortened N = 276	Maintained N = 192	Lengthened N = 140	P
Age	67±11	65±10	66±10	0.39
Male (%)	241(87)	145(76)	113(81)	0.004
Baseline Telomere Length (T/S)	1.06±0.19	0.86±0.15	0.71±0.14	<0.001
Ethnicity (%)				0.20
- Hispanic	20 (7)	19 (10)	19 (14)	
- Asian	28 (10)	26 (14)	19 (14)	
- Black	55 (20)	26 (14)	17 (12)	
- White	167 (61)	115 (60)	80 (57)	
- Other	6 (2)	6 (3)	5 (4)	
Education > High School (%)	199(72)	137(71)	108(77)	0.49
Income ≥ $50000 (%)	56(20)	49(26)	32(23)	0.1
Hypertension (%)	200(73)	130(68)	95(68)	0.45
Prior MI (%)	146(53)	93(48)	78(56)	0.39
Prior CHF (%)	44(16)	26(14)	20(15)	0.77
Prior Stroke (%)	30(11)	23(12)	22(16)	0.35
Type II Diabetes (%)	62(22)	49(26)	35(24)	0.71
Prior Revascularization (%)	175(63)	121(64)	70(50)	0.02
Current Smoking (%)	50(18)	26(14)	25(18)	0.38
Past Smoking (%)	144(52)	95(50)	68(49)	0.74
LVEF	0.63±0.09	0.62±0.09	0.61±0.09	0.06
BMI	28±5.1	28±5.0	29±5.4	0.56
Waist-to-hip ratio	0.96±0.08	0.94±0.08	0.95±0.08	0.003
Physically Active (%)	190(69)	131(69)	90(64)	0.59
Exercise Capacity (Mets)	8.1±3.2	8.1±3.1	7.5±3.4	0.19
Statin use (%)	187(68)	140(73)	96(69)	0.47
Beta-blocker use (%)	161(58)	113(59)	77(55)	0.75
ACE/ARB use (%)	140(51)	100(52)	69(49)	0.88
Aspirin use (%)	227(82)	147(77)	101(72)	0.05
Vitamin use (%)	73(27)	31(16)	24(17)	0.01
Log Fasting Glucose	4.7±0.2	4.7±0.3	4.7±0.2	0.85
Log HDL	3.8±0.3	3.8±0.3	3.8±0.3	0.27
Log LDL	4.6±0.3	4.6±0.3	4.6±0.3	0.14
Log CRP	0.58±1.3	0.54±1.3	0.60±1.3	0.89
Log Insulin	5.6±0.7	5.7±0.6	5.7±0.5	0.30
Log TNF-alpha	1.2±0.8	1.2±1.0	1.1±0.9	0.71
Log IL-6	0.9±0.7	0.8±0.7	0.9±0.7	0.61
Log Adiponectin	16.8±0.8	16.9±0.8	16.8±0.7	0.55
Log Leptin	8.9±1.1	9.0±1.0	9.1±1.1	0.18

We then used multivariable logistic regression models to identify the independent predictors of leukocyte telomere shortening (vs. maintained or lengthened) as a dichotomous variable, again using a stepwise backward selection strategy with predictors retained in the final model at p<0.1. Model adequacy was confirmed using the Hosmer-Lemeshow goodness of fit test. Statistical analysis was performed using Intercooled STATA 10.0 (STATA Corporation, College Station, TX). The authors take responsibility for the integrity of the data. All authors had full access to the data, and have read and agree to the manuscript as written.

## Results

**Figure 1:**
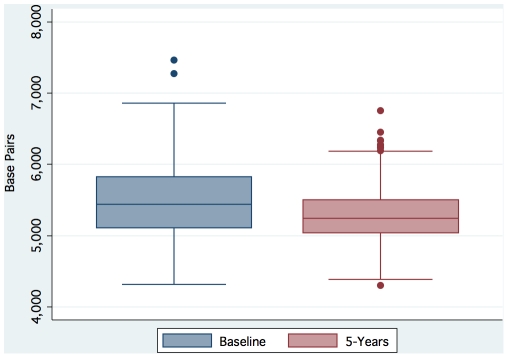
Distributions of baseline and follow-up leukocyte telomere length.

Baseline telomere length, follow-up telomere length, and change in telomere length were all normally distributed (**Figures 1** **and** **2**). The mean (±SD) leukocyte telomere length at baseline was 5496 (±528) base pairs. The mean (±SD) leukocyte telomere length after 5 years of follow-up was 5286 (±355) base pairs. The mean telomere length decreased by 42 base pairs per year (p<0.001). Overall, telomere shortening was observed in 45% of participants; telomere maintenance was observed in 32% of participants; and telomere lengthening was observed in 23% of participants. Hence we use the term telomere trajectory to emphasize that leukocyte telomere length in humans does not inevitably decrease over time.

**Figure 2:**
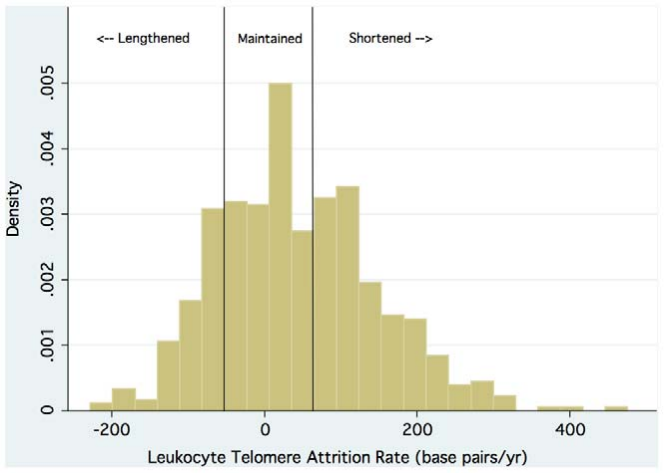
Distribution of leukocyte telomere attrition rate (base pairs per year).

The characteristics of the study population categorized by leukocyte telomere trajectory are shown in **Table 1**. In unadjusted bivariate analyses, participants whose telomeres shortened were more likely to be male, had longer baseline telomere length, greater waist-to-hip ratio, higher prevalence of prior revascularization, and more multivitamin use than participants whose telomere length was maintained or increased. There were no significant differences in ethnicity, education, income level, body mass index, co-morbidities, or smoking across categories of telomere trajectory.

**Figure 3:**
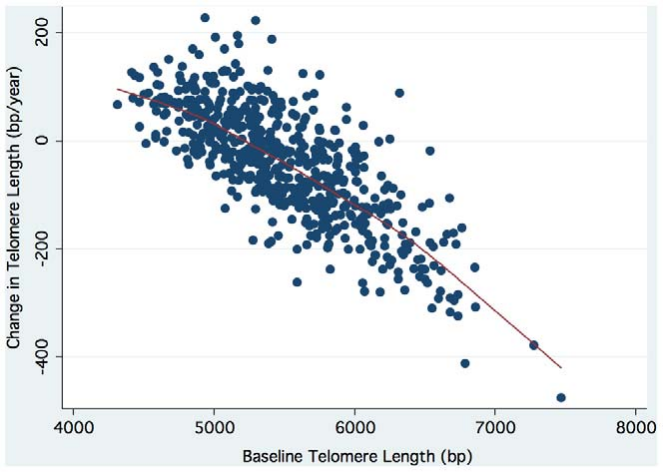
Scatterplot of change in telomere length against baseline telomere length (r = 0.76; p<0.0001).

The independent predictors of change in leukocyte telomere length retained in the final linear regression model were: baseline telomere length (T/S), the square of baseline telomere length (T/S)^ 2^, age, male sex, and waist-to-hip ratio. The beta-coefficients and corresponding 95% confidence intervals for each independent predictor are summarized in **Table 2**. The inverse association of change in telomere length with baseline telomere length is illustrated in **Figure 3**.

**Table 2:** Independent predictors of change in leukocyte telomere length as a continuous variable (multivariable linear regression with backward selection of candidates in table 1 retained at p<0.1).

Variable	Beta-coefficient (base pairs/year)	95% CI	P value
Baseline Telomere Length (T/S)	−151	−302, +1	0.05
Baseline Telomere Length (T/S)^2^	−108	−185, −30	0.007
Age (per 10 yrs)	−16	−21, −11	<0.001
Male	−25	−40, −10	0.001
Waist-to-hip ratio	−86	−156, −16	0.02

Final model also adjusted for ethnicity and LVEF.

The independent predictors of leukocyte telomere shortening retained in the final logistic regression model were: baseline telomere length (T/S) (OR per SD = 7.6; 95% CI 5.5, 10.6), age (OR per 10 year increase = 1.6; 95% CI 1.3, 2.1), male sex (OR = 2.4; 95% CI 1.3, 4.7), and waist-to-hip ratio (OR per 0.1 increase = 1.4; 95% CI 1.0, 2.0). The odds ratios and corresponding 95% confidence intervals for each independent predictor are summarized in **Table 3**.

**Table 3:** Independent predictors of leukocyte telomere shortening as a dichotomous variable (multivariable logistic regression with backward selection of candidates in table 1 retained at p<0.1).

Variable	Odds Ratio for Telomere Shortening	95% CI	P value
Baseline T/S (per SD)	7.6	5.5, 10.6	<0.001
Age (per 10 yrs)	1.6	1.3, 2.1	<0.001
Male	2.4	1.3, 4.7	0.007
Waist-to-hip ratio (per 0.1 increase)	1.4	1.0, 2.0	0.04

Final model also adjusted for ethnicity and LVEF.

With body mass index, adipokines, and mediators of systemic inflammation forced into a multivariable linear regression model with waist-to-hip ratio, we observed no significant attenuation of the effect of abdominal obesity on telomere shortening (**Table 4**).

**Table 4:** Effect of waist-to-hip ratio on change in telomere length (as a continuous variable) after adjustment for body mass index, adipokines, and inflammatory markers.

Variable	Beta-coefficient* (base pairs/year)	95% CI	P value
Waist-to-hip ratio	−120	−201, +40	0.003
BMI	+1	−1, +2	0.39
Log Adiponectin	−2	−9, +5	0.59
Log Leptin	+5	−1, +11	0.12
Log CRP	+2	−3, +6	0.52
Log IL-6	−10	−19, +1	0.02
Log TNF-alpha	−1	−6, +5	0.83

* Also adjusted for baseline T/S, baseline T/S^2^, Age, Sex, Ethnicity, and LVEF.

## Discussion

In this longitudinal study of 608 patients with stable coronary artery disease, we observed three distinct telomere trajectories: 45% exhibited telomere shortening, 32% maintained the same telomere length, and 23% lengthened their telomeres during 5 years of follow-up. We also identified four independent predictors of telomere shortening in patients with coronary artery disease: longer baseline telomere length, older age, male sex, and higher waist to hip ratio. These observations suggest bidirectional regulation of leukocyte telomere length, and raise the possibility that telomeric aging may be reversed.

Prior studies have observed telomere lengthening in approximately 12–24% of healthy individuals [18], [19], but the trajectory of telomere length has not previously been evaluated in patients with coronary artery disease. Given the physiological burden of chronic illness, one might expect telomere shortening to occur in a greater proportion of patients with coronary artery disease. However, less than half of our sample experienced telomere shortening, and almost a quarter actually lengthened their telomeres during the 5-year follow-up. Moreover, individuals with the longest telomeres experienced the greatest amount of shortening, while those with shorter telomeres either maintained or increased their length. These results demonstrate for the first time that the inverse association previously seen in healthy individuals [18], [19], [20] extends to patients with coronary artery disease. Consistent with mathematical models of telomere shortening [21], these observations further suggest that there may be negative feedback regulation of leukocyte telomere length in humans. An intriguing candidate for mediating such regulation is the enzyme telomerase, which is active in hematopoietic stem and progenitor cells, and at low levels in peripheral blood leukocytes [22].

Surprisingly, once baseline telomere length was taken into consideration, only a few other candidate variables retained an independent effect on telomere trajectory. Specifically, and in contrast with prior cross-sectional studies [12], [13], [23], [24], [25], [26], [27], we found no significant associations between telomere trajectory and ethnicity, socioeconomic status, blood pressure, insulin resistance, smoking, body mass index, physical activity, or consumption of multivitamins. These findings suggest that the epidemiology of longitudinal telomere dynamics is fundamentally distinct from the epidemiology of cross-sectional telomere length. Although many variables may be associated with cross-sectional telomere length at a single time point, reflecting the cumulative lifetime burdens of genetic and environmental exposures, most of these effects appear to be dwarfed by the apparent negative feedback from baseline telomere length to telomere trajectory. Moreover, confounding by measured or unmeasured factors, may have contributed to associations of lifestyle factors with cross-sectional telomere length in previous studies.

In this study of individuals with established coronary artery disease, increasing age and male sex were associated with accelerated telomere attrition, even after accounting for differences in baseline telomere length. These findings conflict with prior studies of healthy individuals, which have reported no significant independent effect of sex[18], and a bimodal effect of increasing age[28]. Further studies are needed to examine the effects of age and gender on telomere maintenance and telomerase activity in persons with and without cardiovascular disease.

The association of obesity with short telomere length has been observed [13], [29], but the effects of body mass index and waist-to-hip ratio on telomere dynamics have not been previously reported. We found no independent association of body mass index with telomere trajectory, suggesting that the biological effect is driven primarily by abdominal obesity. Furthermore, we observed no attenuation of this association after adjustment for adipokines and systemic inflammatory mediators that are typically associated with abdominal obesity. Indeed, abdominal obesity appeared to have an even stronger effect on telomere shortening after adjusting for these variables.

Oxidative stress may provide a potential link between abdominal obesity and telomere shortening. Epidemiologic studies have demonstrated a robust association between abdominal obesity and oxidative stress[30]. Excess production of free radicals causes premature cellular senescence and accelerates age-associated tissue damage in animal models of visceral obesity[31]. Moreover, oxidative stress directly exerts a negative effect on telomere length maintenance, both through inhibition of telomerase activity[32] and direct erosion of GGG triplets in telomeric DNA[33]. Further studies are warranted to elucidate the mechanisms whereby abdominal obesity accelerates telomeric aging in vivo.

No cardioprotective medications were found to have an independent effect on telomere trajectory. In particular, we found no association of statin use with change in telomere length. These findings support and extend the prior observation that, while statins attenuate the excess risk of coronary events conferred by short telomeres, their use is not associated with longer telomeres[7].

Among the strengths of the present study is the measurement of a wide range of candidate variables that have been associated with telomeric aging. The longitudinal study design allowed us to identify the determinants of leukocyte telomere trajectory in a large cohort of patients with stable coronary artery disease. However, several limitations should be considered in the interpretation of our results. First, our measurements were restricted to telomere length in circulating leukocytes and do not necessarily reflect telomere trajectory in other cell compartments such as myocardium, endothelium, or the atherosclerotic plaque. Second, the quantitative PCR technique employed in this study measures the mean telomere length across all chromosomes present in the participant's blood sample. However, evidence from rodents suggests that the shortest telomere, rather than the mean telomere length, may be the more important determinant of cell viability and chromosomal stability[34]. As such, our use of mean telomere length could have resulted in a loss of precision with regard to ascertainment of the shortest telomere length in each cell. Third, we were not able to perform assays of telomerase activity, which might further clarify the mechanisms of telomere lengthening in a subset of patients. Fourth, our evaluation of abdominal obesity was based on waist-to-hip ratio, but more sophisticated imaging techniques such as computed tomography would likely provide more accurate classification in this regard. Fifth, the study sample consisted only of individuals with established coronary artery disease. As such, our findings may not be applicable to healthy individuals, and we are not able to directly estimate the effect of coronary artery disease on the rate of telomere length change.

Finally, as with any repeated measurement of a continuous variable, the possibility of regression to the mean should be considered. This is however extremely unlikely, because baseline telomere length was not used as an inclusion criterion. Indeed, each and every participant with baseline and follow-up telomere measurements was included in the study. Moreover, we measured baseline and follow-up telomere length in duplicate or triplicate for every sample, thereby limiting the effects of random fluctuation due to test conditions. The coefficients of variability achieved approach those of the Southern blot technique, which remains the gold standard for telomere length assessment.

In summary, we report that leukocyte telomere trajectory in a cohort of persons with coronary artery disease is powerfully influenced by baseline telomere length in a pattern suggestive of negative feedback regulation. Age, male sex, and abdominal obesity also independently predicted telomere shortening. Future studies will further elucidate the mechanisms, significance, and reversibility of telomeric aging in cardiovascular disease.
